# Respectful Children’s Shoes: A Systematic Review

**DOI:** 10.3390/children11070761

**Published:** 2024-06-23

**Authors:** Pilar Alfageme-García, Sonia Hidalgo-Ruiz, Sergio Rico-Martín, Julián Fernando Calderón-García, Víctor Manuel Jimenez-Cano, Juan Francisco Morán-Cortés, Belinda Basilio-Fernández

**Affiliations:** 1 Department of Nursing, University Centre of Plasencia, University of Extremadura, 10600 Plasencia, Spain; palfagemeg@unex.es (P.A.-G.); victormajc@unex.es (V.M.J.-C.); bbasfer@unex.es (B.B.-F.); 2 Department of Nursing, Nursing and Occupational Therapy College, University of Extremadura, 10003 Cáceres, Spain; sergiorico@unex.es (S.R.-M.); jfcalgar@unex.es (J.F.C.-G.)

**Keywords:** shoes, children, pediatrics, children’s footwear, respectful children’s shoes

## Abstract

Background: Child footwear, both in pathologies and in normal situations, can affect the foot in various ways depending on its characteristics. Below, some features of child footwear are described, and how they can influence the foot, including suitable size, shape and design, flexibility, and transpirable material; inadequate footwear includes situations with flat foot, equine foot, and hammer toes. It is important to highlight that each child is unique and may have different footwear needs. In case of specific pathologies or concerns, it is recommended to consult a specialist in podology or foot medicine for personalized assessment and recommendations. Methods: The present systematic review was conducted in accordance with the guidelines outlined in the Preferred Reporting Items for Systematic Reviews and Meta-Analyses (PRISMA) statement. Results: Children’s footwear must adapt to all stages of children’s growth, starting from when they begin to walk, to promote the correct evolution of their musculoskeletal system. For up to six months, they do not need to wear shoes; socks and similar clothing are enough to warm your feet like a second skin. The flexibility of respectful footwear is essential between six months and three or four years. From that age onwards, the soles can be somewhat thicker, and the buttress can have a certain firmness, but the shoes should remain flexible. Conclusions: Eco-friendly footwear, which typically comes from small businesses and factories, is sometimes described as “ergonomic footwear”. However, there is some reluctance towards this term. When choosing this type of footwear for children, it is important to not just look at the label; rather, one should verify that it meets all the necessary characteristics to be considered respectful.

## 1. Introduction

Child footwear, both in pathologies and in normal situations, can affect the foot in various ways depending on its characteristics. Some features of child footwear and how they can influence the foot are described below [1,2,3,4,5]:

- Suitable size: Shoes should be the right size for the child. If the shoe is too small, it can cause compression and deformities in the toes, such as hammer toes or bunions. On the other hand, if the shoe is too large, it can make it difficult for stability and balance when walking [6].
- Shape and design: The shoes must have a shape that fits the anatomy of the child’s foot. It should allow for sufficient space for the toes and have a rounded tip that avoids compression and deformation of the toes. Shoes with narrow or pointy tips should be avoided because they can cause problems in the toes [3].
- Flexibility: Children’s shoes should be flexible enough to allow the natural movement of the foot when walking. A flexible sole allows the muscles of the foot to be properly strengthened and contributes to the development of a healthy walk. If the shoe is too rigid, it can limit movement and affect the biomechanics of the foot [4,7].
- Transpirable material: The shoe’s material must be transpirable to allow proper ventilation and avoid moisture accumulation. This helps prevent the proliferation of bacteria and fungi, and reduces the risk of infections and dermatological problems [4].

As for pathologies, some conditions that may be affected by inadequate footwear include the following [8,9,10]:

- Flat foot: Wearing shoes without proper support can aggravate flat feet in children, as it does not provide the necessary bow to hold the foot [5,11,12].
- Equine foot: Inadequate footwear can exacerbate equine standing position (toes pointing down), which can affect walking and balance [13].
- Hammer Toes: Wearing shoes with narrow tips or with little toe space can contribute to the development of hammer toes, where the toes bend in the joint [14].

It is important to highlight that each child is unique and may have different footwear needs. In case of specific pathologies or concerns, it is recommended to consult a specialist in podology or foot medicine for personalized assessment and recommendations [15].

In this sense, respectful children’s footwear refers to shoes designed and manufactured considering the health and well-being of children, as well as the environmental and ethical impact of their production. When choosing respectable children’s shoes, the following characteristics should be considered [16]:

- Natural and sustainable materials: Choosing footwear made from natural, sustainable materials can reduce the environmental impact. Some examples of respectful materials are vegetable leather, organic cotton, linen, or cork [16].
- No toxic substances: It is important to choose shoes free of toxic materials such as lead, phthalates, or azoic dyes, which can harm children’s health [15].
- Flexibility and freedom of movement: Respectful footwear should allow natural movement of the foot and have a flexible sole. This promotes healthy foot development and contributes to proper walking [14].
- Ergonomic design: The shoe design should be appropriate to the anatomy of the child’s foot, with enough space for the toes and a shape that does not compress or restrict movement [13].
- Ethical manufacturing: The origin of footwear must be considered and ensure that it has been produced under fair and ethical working conditions. Find brands that follow responsible production standards and care about the well-being of workers [10].
- Durability: Choosing quality and durable shoes can reduce the need to replace them frequently, reducing the environmental impact [5].

When choosing respectful children’s shoes, it is recommended to research and look for brands that align with these principles. In addition, it is important to consider the individual needs of each child, such as the type of foot, the activities they perform, and their comfort when wearing shoes [17,18].

Types of Shoes According to Child Development [12,19,20]

- Baby shoes (0–6 months, pre-crawling stage of development).

They are not required, and have the exclusive function of protecting against cold, moisture, and shocks. These are the same functions that a sock can perform, preferably without seams. It is up to the parents to choose whether to use one or the other. In the case of choosing shoes, they must have the following characteristics:

- The tip should be round or square, viewed from above and rounded by the side.
- The cutting material should be very flexible.
- It is recommended to use a type of closure with a single loop or Velcro. In order for the child not to lose the shoe, the back should be high, very flexible, and soft.
- The soles should have smooth, soft skin or fabric.
- The interior of the shoe should be like a glove and have a soft finish and no seams
- Shoes for infants (6–18 months, crawling stage of development).

Their function, like the previous one, is to protect against cold or surfaces (if this is not the appropriate one), and in other cases, they are decorative. Otherwise, walking without shoes is crucial for the normal psychomotor development of the child and the strengthening of the foot. Likewise, if you want to opt for shoes, the following characteristics are recommended:

- The pointer should be round or square from the top and rounded by the side.
- The cutting material should be very flexible.
- It is recommended to use a concorded-type closure with a single lace or Velcro. The rear can be high or folded, and it should be very flexible and soft so that the child does not lose the shoe.
- The soles should be smooth, sliding proof, 2 or 3 mm soft rubber.
- The interior of the shoe should be like a glove, with a soft finish, and seamless.
- Shoes for beginners (1.5–3 years, stage of acquisition of walking).

The child stands up and takes his first steps. At first, he has an irregular walk with trouble keeping balance. For the above, it is recommended to choose shoes with the following characteristics:

- The inner hole in the length should be about 10 mm.
- The pointer should be round or square, viewed from above, and rounded by the side. It should be closed with a reinforcement of some stiffness to protect the toes.
- The cutting material should provide flexibility, protection against cold, and allow sweating. The wrapping should be high on the embroidery with a soft leather tongue.
- A soft and flexible shoe is recommended, with soft adjustment to keep the heel inside the shoe.
- The soles should be flat (no more than 3 mm). It should not be very soft but very flexible in the toes and with moderate friction characteristics. If you have a heel, the maximum height should be between 3 and 5 mm.
- The coating must have a certain grip to avoid slipping of the foot and the shoe. The interior should be soft, without inner seams.
- Children’s shoes (4–7 years old, walking maturity stage).

It is a period of acquisition and maturation of the march. The activity of the child requires shoes, and they will have to protect the foot from possible injuries, so they should have the following characteristics:

- The inner hole in the length should be between 10 and 15 mm.
- The pointer should be round or square, viewed from above, and rounded by the side. It should also be closed with reinforcement of some rigidity for the protection of the toes.
- The cutting material should provide flexibility, protection against cold, and allow for sweating.
- The embroidery should be high on the pin with a soft leather tongue. A Velcro-type easy-to-use closure is recommended.
- The soles must be flexible. It must have a continuous thickness between 5 and 10 mm and be of a material not too hard, with damping properties. The maximum height of the heel should be between 5 and 10 mm. Materials such as rubber and polyurethane can provide the right characteristics.
- It is recommended to include a firm counterport without becoming completely rigid.
- Shoes for children (7–14 years old, stage of increased physical activity).
- The height of the heel should not exceed 10 mm in children from 7 to 10 years of age. Between 10 and 14 years of age, this height should not exceed 15 mm in boys and 20 mm in girls.
- The pointer should be round or square, seen from above, and rounded by the side. It must also be closed with a reinforcement of some rigidity for the protection of the toes.
- The cutting material should provide flexibility, protection against cold, and allow sweating.
- It is convenient that the embroidery is high on the stitch with a soft leather tongue.
- It is advisable to incorporate a minimum thickness of 15 mm with good absorption properties and resistance to abrasion.
- The coating must be rough in the counterport area to prevent the shoe from loosening.

As to the most effective characteristics that shoes should have, the following are listed. 1. It should be quadrangular to form the normal foot configuration, with plenty of space for the toes. 2. It should be flexible to allow free movement of the foot. 3. It should be a flat plane, without height lifting, and porous, with the top made of unsealed leather or fabric to avoid maceration of the skin or mycotic infections. 5. It should provide moderate traction; the friction of the soles should be equivalent to the bare foot. Soles that are slick (skin) or that create excessive friction (some rubber soles) should be avoided. 6. It should be lightweight to reduce energy expenditure. 7. Part of the shoe should cover the ankle in the older infant to prevent the shoes from falling off when running. 8. The appearance should be acceptable, as children are very sensitive about this. 9. It should be a reasonable price; a medically acceptable shoe should not be expensive. Tennis shoes are often recommended for children. Unfortunately, they comprise a wide variety of types; some are ideal, fulfilling the criteria perfectly, while others have rigid soles with occlusive tops. Doctors should describe the proper characteristics of the tennis shoe to parents. In the future, shoes will have to be designed to maximize function and provide comfort and protection. The routine incorporation of maximum absorption features into the shoe will decrease the incidence of common overuse syndromes during late childhood and adolescence [21,22,23,24,25].

This study aims to understand the importance of child-friendly footwear.

## 2. Materials and Methods

### 2.1. Study Design

The systematic review was conducted following the guidelines set out in the PRISMA (Preferred Reporting Items for Systematic Reviews and Meta-Analyses) statement [26]. The review protocol is registered in PROSPERO with the number “CRD42024492728”, which allows access to the relevant articles. A comprehensive search was carried out in various databases, including PubMed, Cochrane, Dialnet, Scopus, Web of Science, PsycINFO, and Science Direct PEDro (Physiotherapy Evidence Database) (RISMA Checklist in Supplementary S1).

### 2.2. Search Strategy

Keywords were used in the abstract and title fields, such as “Shoes”, “Children”, “Pediatrics”, “Children’s Footwear”, and “Respectful Children’s Footwear”. These keywords were entered in Spanish when necessary, according to the database. Spanish terms were also used, such as “Zapatos”, “Children”, “Pediatrics”, “Children’s Footwear” and “Respectful Children’s Footwear”. Keywords were combined using the Boolean operators AND or OR. The syntax of the descriptors combined in the search in scientific databases is detailed in Table 1.

### 2.3. Inclusion and Exclusion Criteria

The following inclusion criteria were applied in this study: (a) controlled (C) and uncontrolled (NC) clinical trials were included; (b) the articles must have been published in the last 12 years; (c) articles written in English or Spanish were accepted; (d) individuals 1 month of age or older were considered. The search was restricted to the last 12 years to examine the most recent advances in the use of virtual reality in the analyzed variables and update the scientific evidence available in the literature on this topic [4,7].

The exclusion criteria were established following the PICO model (population, intervention, control, comparison, and results). The exclusion criteria were as follows. Literature reviews and any type of document that was not a clinical trial were excluded; studies that used techniques other than children’s footwear were excluded; and treatments performed in patients under 1 month of age were excluded.

### 2.4. Study Selection

**Figure 1:**
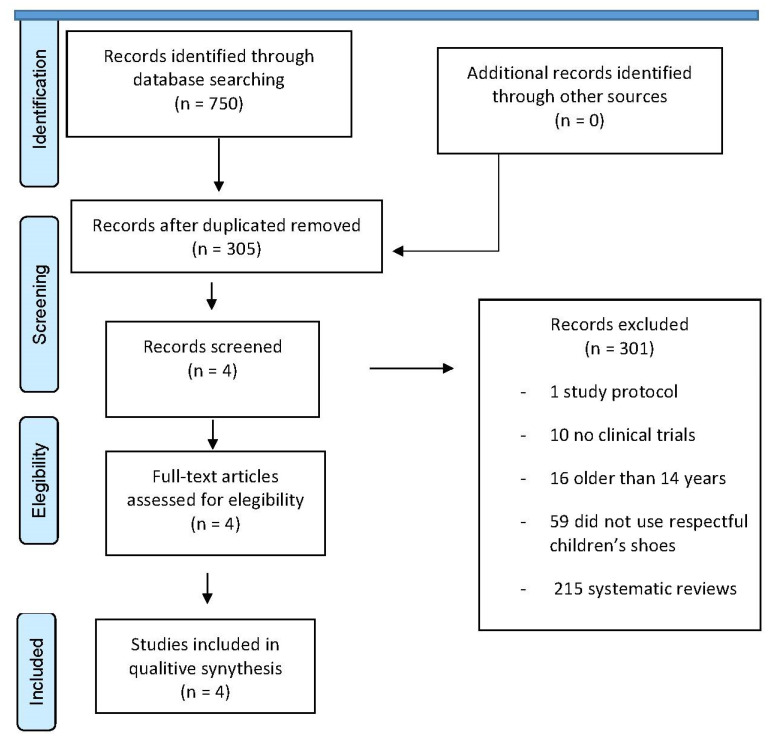
PRISMA flowchart.

A preliminary screening of publications was conducted, considering their relevance to the chosen research topic. The process of selecting studies involved a thorough examination of their abstracts, with the exclusion of those that did not satisfy the predetermined criteria. The whole texts of the papers that satisfied the inclusion criteria were thoroughly examined, analyzed, and incorporated into the systematic review. The two reviewers independently retrieved and examined all potential full-text publications. While the specific calculation of agreement between the two reviewers was not conducted, any discrepancies about the inclusion or exclusion of full-text publications were handled by conversation, as depicted in Figure 1.

The data collected for this review encompassed various elements, including the author and date of each study, details about the study sample such as sex and mean age, the inclusion and exclusion criteria employed, the intervention implemented, the duration of the follow-up period, the evaluation scales utilized, and the results achieved from each study. The data were compiled in a conventional tabular format. The reviewers responsible for article selection additionally conducted independent data acquisition and evaluated the methodological rigor of the investigations. In the event of any divergent viewpoints, they were effectively addressed through the process of deliberation and dialogue.

### 2.5. Assessment of Methodological Quality

To assess the risk of bias, the ROBINS-1 tool was used. A qualitative review of the data was performed to assess heterogeneity and bias. Evidence tables (study characteristics and outcomes) were generated, and quantitative synthesis was performed if data were homogeneous.

ROBINS provides signaling questions whose answers indicate the potential for bias, thus providing a systematic way to organize and present the available evidence related to the risk of bias in NSAs. Depending on the responses to the signaling questions, the options for each domain are low, moderate, severe, or critical risk of bias, with an additional option: no information.

### 2.6. Risk of Bias Analysis

The assessment of bias was conducted for each study that was included, encompassing various types of bias such as selection bias, performance bias, detection bias, attrition bias, reporting bias, and other potential biases. In this evaluation, seven categories were assessed. 1. The first factor to consider is random sequence generation, which may introduce selection bias. 2. Another important factor is allocation concealment, which can also lead to selection bias. 3. Additionally, the blinding of participants and personnel is crucial to minimize performance bias. 4. Furthermore, the blinding of outcome assessment is essential to reduce detection bias. 5. Incomplete outcome data should be carefully addressed to avoid attrition bias. 6. Selective reporting should be avoided to prevent reporting bias. 7. Lastly, it is important to consider any other potential biases that may arise in the study. The assessment of bias and study quality was conducted by a single reviewer.

## 3. Results

The literature search was conducted in May 2023. A total of 705 studies were obtained from the search in all databases. The PRISMA flowchart (Figure 1) shows the selection process of the studies. The records that were duplicated were excluded, and 305 records were screened. Finally, four studies were included in this review. Table 2 shows the main findings of this review.

After a thorough analysis of the 305 records, those that met the criteria established for this review were included. In this sense, only four studies that were considered relevant and of quality were selected.

It is important to note that this review focuses on non-randomized studies, which provide relevant information on the topic of healthy children’s shoes. The results obtained from these four included studies are presented in Table 2, which summarizes the main findings found in this review.

These results demonstrate the importance of carrying out an exhaustive and selective review, with the aim of obtaining quality and reliable information on the topic in question.

### Description of the Results

The main characteristics of the studies are shown in Table 2. The most relevant aspects are the following:

Sample: N = 23 [27], N = 33 [28], N = 102 [29], N = 100 [30].

We found a higher frequency of men than women [28,30] among the sample. Only two studies [27,29] had a sample where the female sex prevailed over the male sex but without a significant difference.

Methodological quality: Table 3 displays the outcomes of the evaluation conducted to determine the methodological quality. It is important to acknowledge that a negative response does not automatically imply the absence of a certain quality in the study. Rather, it indicates that the requirement was not identified in the text, even after conducting a comprehensive examination of the article.

According to the evaluation carried out using the Physiotherapy Evidence Database (PEDro) scale, all included studies obtained scores indicating good methodological quality, with scores of 7 and 8 [27,28,29,30]. This indicates that the studies meet a series of important criteria to guarantee the validity and reliability of the results obtained.

## 4. Discussion

The following section presents the most pertinent and definitive findings from each study, as an illustrative example.

The pragmatic study’s objectives of examining the factors influencing these kids’ footwear preferences and supplying data on their impression of comfort and vocabulary related to shoes have been met. It has also exposed disparities concerning particular age cohorts. This study shows that, in accordance with developmental theory, any comfort measurement tool must be age group-specific, even though it offers crucial information as a starting point for creating one. Overall, every youngster in the survey consistently used the adjective “softness” to describe comfort. Footwear selection, however, is shown to be a multifaceted concept influenced by practical considerations, psychological aspects, identified “discomfort” in particular shoe locations, and aesthetics. Parental or guardian involvement is valuable as well, as it greatly promotes autonomy and choice [27].

There has not been a consensus on how to describe and characterize clinical footwear therapies for children with mobility impairments, despite their historical and widespread use. Together with previously available data, new concepts were able to be synthesized thanks to the expert panel’s consensus and the inductive, iterative approach of this study. A unified understanding of clinical footwear interventions for children with mobility limitation led to their collective grouping and definition under the general term therapeutic footwear. This made it possible to identify and classify stability footwear as a subgroup of functional footwear, which is one of these interventions with the highest potential for effectiveness. A shared knowledge of the best design features for readily available stability therapy footwear and its potential applications for a variety of pediatric mobility disabilities was also produced by the process. As previously mentioned, just one study examined professional opinions regarding footwear as a pediatric therapeutic intervention. The current study has identified the particulars and goal of off-the-shelf stability therapeutic footwear design as well as criteria for clinical prescription for children. It has also provided a more thorough synthesis of expert opinion offering consensus on terms and definitions for children’s clinical footwear interventions [28].

No child at Miguel de Cervantes School wore a shoe that was appropriate for his or her daily activity and age. A total of 0% of the participants wore a shoe that was within the normal range for their fullness. A total of 38% of the participants wore shoes with a length that was appropriate for their age and daily activity according to the literature. Eighty-nine percent of participants wore shoes where the widest area of the shoe coincided with the metatarsal heads. A total of 100% of the participants wore shoes with a cut material that matched the climatic conditions according to the bibliography. A total of 99% of the participants wore shoes with a support size according to their age and daily activity according to the literature. A total of 5% of the participants wore shoes with a toe cap according to their age and daily activity according to the literature. A total of 54% of the participants wore shoes with a rearfoot size appropriate to their age and daily activity according to the literature. A total of 90% of the participants wore shoes with an age- and activity-appropriate toe box according to the literature. A total of 45% of the participants wore shoes with a toe breaker according to their age and daily activity according to the bibliography [29].

The procedure used for the design of children’s footwear is a linear scale from molds taken from adult feet, and the latter constitute the model for the creation of different sizes, without considering the structural differences between the child’s and the adult’s foot. On the other hand, in the stage of increasing walking activity, footwear begins to be more like that of the adult, although it still shares some of the characteristics of the infant stage that should be taken into consideration. Eighty-five percent of the students use specific running-style athletic shoes, which are the most versatile and the most adapted to the activities with adequate flexion, adequate sole angulation, weight, type of closure, and well adapted to the surface they use, although for specific sports such as soccer or basketball, it would be preferable for them to use more specific shoes for those sports [30].

## 5. Limitations of the Study and Further Research

Numerous studies have addressed the importance of respectful infant footwear; there are countless systematic and bibliographic reviews that speak descriptively of this whole process for the child from birth; however, few investigations have specifically and scientifically explored in case controls respectful infant footwear, taking children of pediatric age as a sample.

## 6. Implications of the Use of Respectful Children’s Shoes in Clinical Practice

With everything we have seen so far, we have already been able to get an idea of the importance of using respectful footwear, and even more so if we are talking about footwear for children. The main advantage is that it respects the natural growth of their feet, which will ensure optimal development, especially in the musculature and bone support. This, in turn, will prevent deformities and unnecessary stress, something that is not considered in the manufacture of commercial footwear, causing the development of children’s feet to be totally limited and corseted.

## 7. Conclusions

When we affirm that respectful footwear is more than a fashion, we refer to the need to take care of the development of children’s feet to avoid deformations and injuries. Respectful shoes, unlike others, avoid podiatric health problems.

In no case is it recommended that children use miniature versions of adult footwear, which are usually common in sports shoes and hiking boots from certain brands.

Children’s footwear must adapt to all stages of children’s growth, from when they begin to walk, to promote the correct evolution of their musculoskeletal system. Up to six months, they do not need to wear shoes; socks and similar clothing are enough to warm your feet like a second skin. Between six months and three or four years, the flexibility of respectful footwear is essential. From that age onwards, the soles can be somewhat thicker, and the buttress can have a certain firmness, but the shoes should remain flexible.

Eco-friendly footwear, which typically comes from small businesses and factories, is sometimes described as “ergonomic footwear”. However, there is some reluctance towards this term. When choosing this type of footwear for children, do not just look at the label, but verify that it meets all the necessary characteristics to be considered respectful.

Avoid shoes with narrow toes that prevent the child from extending their toes naturally. Also, reject those that do not offer flexibility around the comb and the front part of the toes. Make sure the shoe has no heel or heel rise, so that the toe and heel are at the same height. Also, remember the importance of choosing the right size, leaving between 1 and 1.5 cm between the tip of the longest toe and the end of the shoe to ensure comfort when walking.

Respectful footwear is essential for the healthy development of children’s feet and should be chosen with attention and care.

## Figures and Tables

**Table 1:** Syntaxes of combined descriptors in the scientific database search.

Database	Syntax Adopted
PubMed	‘Shoes AND Children AND Pediatrics’; ‘Shoes AND Children AND Children’s footwear’; ‘Shoes AND Children’s footwear AND Respectful children’s shoes’
Cochrane	‘Shoes AND Children AND Pediatrics’; ‘Shoes AND Children AND Children’s footwear’; ‘Shoes AND Children’s footwear AND Respectful children’s shoes’
Dialnet	‘Shoes AND Children AND Pediatrics’; ‘Shoes AND Children AND Children’s footwear’; ‘Shoes AND Children’s footwear AND Respectful children’s shoes’
Scopus	‘Shoes AND Children AND Pediatrics’; ‘Shoes AND Children AND Children’s footwear’; ‘Shoes AND Children’s footwear AND Respectful children’s shoes’
Web of Science	‘Shoes AND Children AND Pediatrics’; ‘Shoes AND Children AND Children’s footwear’; ‘Shoes AND Children’s footwear AND Respectful children’s shoes’
PsycINFO	‘Shoes AND Children AND Pediatrics’; ‘Shoes AND Children AND Children’s footwear’; ‘Shoes AND Children’s footwear AND Respectful children’s shoes’
Science Direct	‘Shoes AND Children AND Pediatrics’; ‘Shoes AND Children AND Children’s footwear’; ‘Shoes AND Children’s footwear AND Respectful children’s shoes’
PEDro (Physiotherapy Evidence Database)	‘Shoes AND Children AND Pediatrics’; ‘Shoes AND Children AND Children’s footwear’; ‘Shoes AND Children’s footwear AND Respectful children’s shoes’

**Table 2:** Characteristics of the studies.

Authors	Objective	Sample, Gender, and Mean Age	Type of Study and Intervention	Treatment and Follow-Up Period	Duration	Results
Price et al., 2021 [27]	Investigate these three factors in order to provide guidance for the creation of a scale for gauging children’s footwear comfort.	N = 23 Sex = child 40%, girl 60%. Mean Age = 1–12 years.	A pragmatic qualitative design with thematic analysis as an analytical approach was implemented	The analytical method used was a pragmatic qualitative design with thematic analysis. A total of 23 kids (ages 1 to 12) were observed passively and briefly interviewed at the headquarters and retail location of a footwear manufacturer. Shoes were to be tried on, and field notes about verbal and nonverbal communication were to be taken. Themes were found, examined, and given names after field notes were coded.	1 week.	In general, the kids equated softness with comfort. But there were many factors that affected the choice of footwear, such as aesthetics, psychological factors, areas of “comfort” and “discomfort,” practical considerations, and predictive worries, all of which interacted with the child’s age.
Hill et al., 2021 [28]	In order to establish a consensus among experts about the definition and grouping of footwear therapies for children, this study focused on the design features and prescription of stability footwear that is readily available for children who have mobility impairments.	N = 33 Sex = child 60%, girl 40%. Mean Age = 1–12 years.	An international expert Delphi consensus study	With round one divided into three sections—terms and definitions, details of off-the-shelf stability footwear design, and criteria for clinical prescription of off-the-shelf stability footwear—a Delphi consensus technique was used. The panel was asked to score how much they agreed with the assertions and to offer more details by asking open-ended questions. The expert opinions were analyzed to evaluate the consensus, which was set at 75% agreement, or to create new assertions that were presented in the next two rounds.	1 week.	It has been determined that the stiffness and width of the sole, along with the heel counter and topline, may have an impact on children’s mediolateral stability during gait. The prescription criteria and outcome metrics for off-the-shelf stability therapeutic footwear for people with Down syndrome, cerebral palsy, Duchenne muscular dystrophy, spina bifida, and mobility symptomatic pes planus have been agreed upon.
Sáez 2019 [29]	Evaluate the condition of the footwear worn by children at the Miguel Cervantes School. Determine whether school-age children 6–8 years old wear footwear that is appropriate footwear for their age and daily activity. Elaboration of an instrument to measure children’s footwear in an interdisciplinary consensus by health professionals. Promotion of podiatric health in the pediatric population.	N = 102 Sex = child 40%, girl 60%. Mean Age = 6–8 years	A descriptive cross-sectional study, in which a bibliographic search was performed in Pubmed, Índice Medico Español (IME), WebOfScience, UpToDate, Scopus and Enfispo. Different searches were performed using as keywords calzado/footwear, niños/children, infancia/childhood, and colegio/school.	The study was carried out on a total of 102 students who completed and submitted the authorization. It was carried out at the Miguel de Cervantes Public School in Elche, Spain. The students had to be within the age range to be studied, from 6 to 8 years old, which corresponded to the first (1ºA and 1ºB) and second (2ºA and 2ºB) grades of primary school.	2 weeks.	– A total of 0% of the participants wore a shoe that was within the normal range for their fullness. A total of 38% of the participants wore shoes with a length that was appropriate for their age and daily activity according to the literature. Eighty-nine percent of participants wore shoes where the widest area of the shoe coincided with the metatarsal heads. A total of 100% of the participants wore shoes with a cut material that matched the climatic conditions according to the bibliography. A total of 99% of the participants wore shoes with a support size according to their age and daily activity according to the literature. A total of 5% of the participants wore shoes with a toe cap according to their age and daily activity according to the literature. A total of 54% of the participants wore shoes with a rearfoot size appropriate to their age and daily activity according to the literature. A total of 90% of the participants wore shoes with an age- and activity-appropriate toe box according to the literature. A total of 45% of the participants wore shoes with a toe breaker according to their age and daily activity according to the bibliography.
Ruiz 2018 [30]	To evaluate whether the sports footwear used by secondary school students in physical education is adequate according to the morphological characteristics of the foot, the surface on which they perform physical and sports activities, in order to determine the consequences of an inadequate use of the same, if any.	N = 100 Sex = child 60%, girl 40%. Mean Age = 12–14 years	Descriptive, observational, and cross-sectional study.			A total of 50% of the schoolchildren have exceeded the time of use of the footwear, 44% wear wider or narrower sports shoes and 51% wear them shorter or longer than they should, 10% have a higher heel than normal and too high or too low. Differences between the heel and forefoot were found below or above the appropriate values for schoolchildren, 58% either do not use counters or they are minimal or too rigid, 92% wear shoes all day or for sports outside the school, 72% have never visited a podiatrist, and 50% of the students buy shoes for the price or design rather than for comfort.

**Table 3:** Physiotherapy evidence database (PEDro) scale.

	Criteria												
Study	1	2	3	4	5	6	7	8	9	10	11	Score	Result
Price et al., 2021 [27]	Y	N	Y	Y	N	N	Y	Y	N	Y	Y	7	GOOD
Hill et al., 2021 [28]	Y	N	Y	Y	N	N	Y	Y	Y	Y	Y	8	GOOD
Sáez [29]	Y	Y	N	Y	N	N	Y	Y	Y	Y	Y	8	GOOD
Ruiz 2018 [30]	Y	Y	Y	N	N	N	N	Y	Y	Y	Y	7	GOOD

Criteria description: 1: Inclusion of clear and appropriate selection criteria; 2: Random assignment; 3. Allocation Hiding; 4. Blinding of participants; 5. Blinding of therapists; 6. Blinding of evaluators; 7. Intention to process data; 8. Comparability of groups at baseline; 9. Follow-up results for at least 85% of participants; 10. Analysis between groups; 11. Results and variability measures reported. Score: Score is the number of Yes (Y) or No (N).

## Supplementary Materials

The following supporting information can be downloaded at: https://www.mdpi.com/article/10.3390/children11070761/s1, Supplementary S1: PRISMA Checklist, Reference [31] cited in Supplementary Materials.
